# A power study of bivariate LOD score analysis of a complex trait and fear/discomfort with strangers

**DOI:** 10.1186/1471-2156-6-S1-S113

**Published:** 2005-12-30

**Authors:** Fei Ji, Dayoung Lee, Nancy Role Mendell

**Affiliations:** 1 Statistical Genetics Laboratory, Rockefeller University, 1230 York Ave, New York, NY 10021 USA; 2 Department of Applied Math and Statistics, Stony Brook University, Stony Brook, NY 11794 USA

## Abstract

Complex diseases are often reported along with disease-related traits (DRT). Sometimes investigators consider both disease and DRT phenotypes separately and sometimes they consider individuals as affected if they have either the disease or the DRT, or both. We propose instead to consider the joint distribution of the disease and the DRT and do a linkage analysis assuming a pleiotropic model. We evaluated our results through analysis of the simulated datasets provided by Genetic Analysis Workshop 14. We first conducted univariate linkage analysis of the simulated disease, Kofendrerd Personality Disorder and one of its simulated associated traits, phenotype b (fear/discomfort with strangers). Subsequently, we considered the bivariate phenotype, which combined the information on Kofendrerd Personality Disorder and fear/discomfort with strangers. We developed a program to perform bivariate linkage analysis using an extension to the Elston-Stewart peeling method of likelihood calculation. Using this program we considered the microsatellites within 30 cM of the gene pleiotropic for this simulated disease and DRT. Based on 100 simulations of 300 families we observed excellent power to detect linkage within 10 cM of the disease locus using the DRT and the bivariate trait.

## Introduction

Due to the complexity of the transmission of complex diseases, more researchers are paying attention to disease-related traits (DRT), or endophenotypes. We define a DRT as an abnormality that 1) appears more frequently in cases (diseased individuals) than in the population, and 2) has a higher frequency in unaffected siblings of cases than in the population. There can be different explanations of the relationship between the disease and the DRT. One explanation is that the traits are determined by a pleiotropic gene, a gene that controls more than one trait. For instance, a single gene mutation may cause an enzyme deficiency, which in turn may affect more than one tissue in one individual [1]. Alternatively a pleiotropic allele may cause both the disease and a DRT abnormality.

The DRT, phenotype b, fear/discomfort with strangers (FDS), appears at a greater frequency in Kofendrerd Personality Disorder (KPD) affected individuals than in the general population. Additionally, the trait FDS appears more frequently in unaffected siblings of KPD individuals than in the general population. Moreover, according to Greenberg [2], both trait and disease are results of an allele at the D1 locus on chromosome 1. Thus, D1 is a major gene for FDS and one of several genes for KPD. We thus consider it to be an ideal candidate for bivariate genetic analysis assuming a pleiotropic model. It turns out that if one is considering a categorical DRT with two outcomes, the bivariate analysis of a disease and DRT reduces to a univariate linkage analysis of a trait with 4 phenotypes. Thus, the extension of standard linkage techniques to a pleiotropic gene is quite straightforward. In this study we report the power of analyses done using this approach in contrast to what one would obtain upon considering the disease and DRT separately.

### Subjects

The study was conducted on nuclear pedigree datasets collected from Aipotu, Karangar, and Danacaa, respectively, and the combination of these three datasets. The simulated data from each city contained 100 nuclear families averaging about 7 members per family. There was no missing phenotype or marker data. Each dataset was simulated 100 times [2].

In order to demonstrate that FDS was a DRT we investigated its distribution in offspring of these nuclear families. For our sample of probands, we used all offspring in these families who had KPD. All offspring who did not have KPD were considered unaffected siblings of probands. Upon doing this we noted that in Karangar 47% of the probands had FDS, 3% of the unaffected siblings of KPD probands had FDS; the rate of FDS in Karangar was 2% [2]. A slightly stronger association between FDS and KPD was observed in Aipotu, with 66% of probands with KPD having FDS, 4% of unaffected siblings of KPD probands having FDS, and a population prevalence of 2%. The strongest association between FDS and KPD was observed in Danacaa, however, where 100% of the probands with KPD had FDS, 8% of the unaffected siblings of probands had FDS, and the population rate was 2%.

### Univariate and bivariate trait linkage analysis

We noted first that the two binary traits could be considered as a single trait with 4 phenotypes, which we defined as follows: 1) KPD positive and FDS positive, KPD+ FDS+; 2) KPD positive and FDS negative, KPD+ FDS- ; 3) KPD negative and FDS positive, KPD- FDS+; 4) KPD negative and FDS negative, KPD- FDS-.

There are then 12 penetrance values in penetrance matrix ψ_**B**_, with entries *g*_*u*_(*x*). Here *g*_*u*_(*x*) denotes the penetrance of the phenotype *x *for the *u*^th ^genotype, for *u *= 1, 2, 3 and *x *= 1, 2, 3, 4, where . A nuclear family dataset is composed of information on family size, the phenotype of interest, and the marker genotype for each individual. We used disease locus allele frequencies, and marker allele frequencies provided by Genetic Analysis Workshop 14 (GAW14) [2].

Conditional on the genetic parameters and the joint distribution of the 4 phenotypes and marker genotypes in family members, we calculated the LOD score for the bivariate trait for a given nuclear family as follows:



Here, as in Elston and Stewart [3], *p*_*stu *_denotes the probability that an offspring has genotype *u *at the disease/DRT locus given that the parents disease/DRT genotypes are *s *and *t*; *g*_*u*_(*x*_*i*_) denotes the probability of having trait phenotype *x *(*x *= 1, 2, 3, 4) given that the disease/DRT genotype is *u *for the *i*^th ^offspring in the nuclear family; *n *denotes the number of offspring; *p*_*fm *_(*p*_*mm*_) denotes the probability that the father's marker (mother's marker) genotype is *fm *(*mm*); *p*_*t *_(*p*_*s*_) denotes the probability that the father (mother) has disease/DRT genotype *s *(*t*).

The algorithm described above was implemented in a C++ program, GAWBI [4,5], which can be used to yield LOD scores of univariate traits and bivariate traits for any arbitrary nuclear family pedigree dataset.

We computed the bivariate LOD score for the combined city samples of 300 nuclear families and the average of these bivariate LOD scores over 100 replicates (bivariate ELOD). This value was then compared to the average LOD score obtained on considering the disease status alone (ELOD-disease) and the trait status alone (ELOD-DRT) using the usual univariate LOD score method and using the marginal penetrance values of KPD and FDS assumed in the bivariate analysis. We then calculated the frequency of the bivariate LOD ≥ 3.0 out of 100 replicates and referred to this value as the bivariate power. Power was similarly obtained for disease and DRT.

We used the gene frequencies given by GAW14 [2] as the values in the analysis model. We did not know the penetrance values for the combined KPD-FDS phenotypes nor did we know the penetrance values for KPD or FDS separately. We read the information on these two traits and used this to make an educated guess as to these penetrance values. In Table 1 we indicate the genotype phenotype penetrance matrix that we used for our analysis.

The markers in the region close to locus D1 contained in the microsatellite files were investigated. The number of alleles at each marker varied from 4 to 9.

## Results

**Figure 1:**
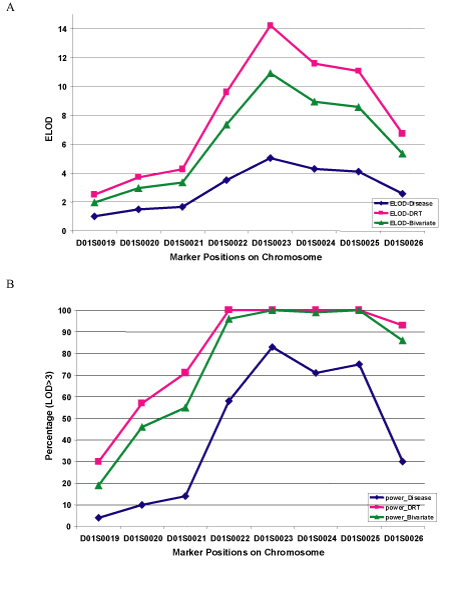
Linkage analysis of disease, DRT, and bivariate trait with the three cities dataset: ELOD (A) and power (percentage (LOD>3)) (B).

The ELOD results for the linkage analysis of the samples of 300 families are presented in Figure 1A. Figure 1A gives the ELOD for considering 1) bivariate analysis of the joint distribution of KPD and FDS, or equivalently a 4-phenotype univariate trait (bivariate ELOD), 2) analysis of trait FDS alone (ELOD-DRT), and 3) analysis of the disease, KPD alone (ELOD-disease). All three approaches show high ELOD values for the markers very close to the susceptibility locus, D1, which is between D01S0021 and D01S0023. The ELOD obtained through analysis of FDS appears to be consistently higher than the ELOD obtained through analysis of KPD alone or through the analysis of the bivariate trait KPD/FDS. The ELOD obtained through analysis of the bivariate KPD/FDS trait is always higher than that obtained through analysis of KPD alone.

In Figure 1B, we present the power to detect linkage using a critical value of observed LOD ≥ 3.0. We observe very high power (>90%) to detect linkage for loci within 10 cM of the KPD/FDS locus D1 on analysis of FDS alone and/or using the bivariate analysis. We also observe essentially no difference in power for analyses based on FDS as compared to KPD/FDS.

**Figure 2:**
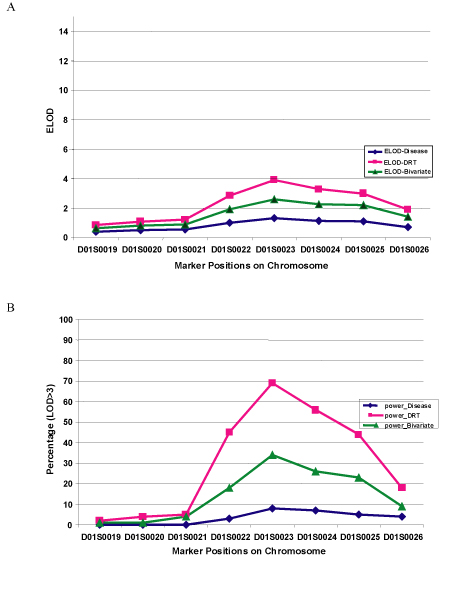
Linkage analysis of disease, DRT, and bivariate trait with the Aipotu dataset: ELOD (A) and power (percentage (LOD>3)) (B).

**Figure 3:**
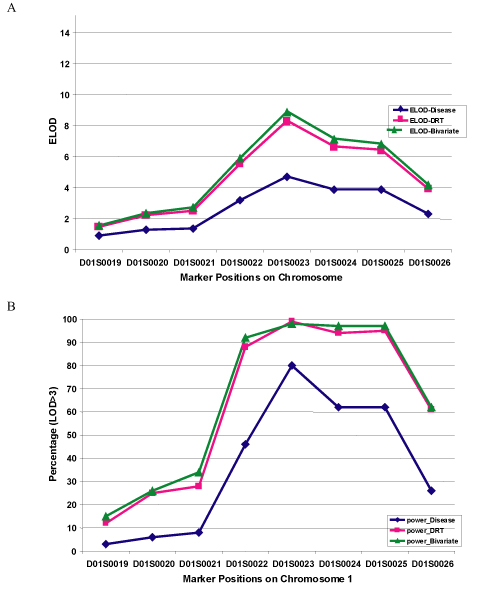
Linkage analysis of disease, DRT, and bivariate trait with the Danacaa dataset: ELOD (A) and power (percentage (LOD>3)) (B).

In Figures 2, we present corresponding ELOD and power results for analysis of 100 replicates of the 100 families in Aipotu dataset. In this case the ELOD, as expected, is much lower than that observed in analyses of the three cities combined. However, it has a similar pattern to that shown in Figure 1A. The corresponding power plot (Figure 3A) shows that considering the DRT alone appears to have greater power than the bivariate approach. Similar results were observed with the Karangar data (not shown). However, the ELOD and power for Karangar data were even lower than the values obtained for the Aipotu data. In Figure 3, we present corresponding results for analysis of 100 replicates of the 100 families in Danacaa. In this case the ELOD and power obtained in the bivariate analysis are the same as those obtained upon the analysis of FDS alone.

In all ELOD and power figures, the highest values are observed at marker position D01S0023, which is the closest marker to D1 (the major susceptibility gene).

## Discussion and Conclusions

The results observed for the linkage analysis of FDS/KPD appear at first to be counterintuitive. We would expect that the bivariate approach would result in greater power than consideration of a single trait. This is indeed what we observed in comparing the two approaches under a wide range of generating models [4]. However Ji [4] considered the situation in which the analysis model parameter values were always correct. There are 12 penetrance values (9 of which are functionally independent) and 1 gene frequency parameter involved. In the case of analysis of a single dichotomous trait there are 6 penetrance values (3 of which are functionally independent) and 1 gene frequency parameter. In this analysis we only have accurate information on the allele frequencies. We also have a situation in which these penetrance values are not the same in all 3 cities so there is not 1 set of correct values for the combined sample of 3 cities.

We did have a rough estimate of the generating model and perhaps did make a better than average guess than one could do in reality. However Ji [4] investigated the robustness of the analysis to using analysis parameter values which were not equal to the correct ones and obtained a slight reduction in power.

Our results indicate that the power of the bivariate analysis appears to be more sensitive to the accuracy of the penetrance values assumed than the analysis of the DRT alone. Ji [4] did note many situations in which analysis based on the DRT was as powerful as analysis based on the bivariate trait. However, she did not observe any penetrance parameter value for which analysis of the DRT was more powerful than analysis of the bivariate trait. In Danacaa the criterion for designating an individual as KPD+ was much narrower than the other two cities, resulting in all KPD+ individuals also being FDS+. This may be a situation where analysis of the DRT is as powerful as the bivariate analysis. Additionally, there was less genetic heterogeneity in the Danacaa sample.

These findings indicate the need to consider several analysis model parameter values with a correction for the number of parameter values considered as is done in LOD score analysis of a single binary phenotype. We also need to realize that there are going to be some situations in which analysis of the trait alone might be as powerful as analysis of the bivariate trait.

The bivariate analysis discussed in this study was done using software (GAWBI) developed by Ji and Yoo [4,5]; however, simple manipulation of the data can allow one to calculate the bivariate trait using LINKAGE [6] with 4 liability classes. In the pedigree input file for the LINKAGE program, we set the affection status of all subjects to 2; that is, every person is affected. The liability class is decided based on the subject's bivariate phenotype *j*, *j *= 1, 2, 3, or 4. In the parameter input file, the penetrances of each liability class are set to equal P(j|genotype), where genotypes are disease/DRT genotypes. A simple dataset was tested using both LINKAGE and GAWBI, and identical results were obtained.

## Abbreviations

DRT: Disease-related traits

FDS: Fear/discomfort with strangers

GAW14: Genetic Analysis Workshop 14

KPD: Kofendrerd Personality Disorder

## Authors' contributions

NRM and FJ conceived the study, participated in its design and coordination, and drafted the manuscript. NRM presented this work. FJ developed GAWBI, the bivariate LOD score analysis program for this study. FJ and DL carried out all the genetic analyses and statistical analyses.

## Figures and Tables

**Table 1:** Penetrances of univariate (KPD, FDS) and bivariate (KPD/FDS) traits

Genotypes	Phenotypes					
	
	KPD+ FDS+	KPD+ FDS-	KPD- FDS+	KPD- FDS-	KPD+	FDS+
AA	0.12	0.08	0.24	0.56	0.20	0.36
AB	0.12	0.08	0.24	0.56	0.20	0.36
BB	0.006	0.004	0.0	0.99	0.006	0.006
